# Predicting intravenous thrombolysis outcomes in acute ischemic stroke using machine learning

**DOI:** 10.3389/fneur.2026.1831043

**Published:** 2026-07-23

**Authors:** Mingyue Jiang, Qiong Yue, Guangwang Zhou, Qian Cui

**Affiliations:** 1 Department of Neurology, The Affiliated Hospital of Xuzhou Medical University, Xuzhou, Jiangsu, China; 2 General Services Office, The Affiliated Hospital of Xuzhou Medical University, Xuzhou, Jiangsu, China

**Keywords:** acute ischemic stroke, intravenous thrombolysis, machine learning, outcome prediction, prediction model

## Abstract

**Objective:**

Intravenous thrombolysis remains a cornerstone intervention for improving clinical outcomes in patients with acute ischemic stroke (AIS). Accurate prediction of poor functional outcomes following thrombolysis is essential for optimizing individualized treatment and guiding clinical decision-making. This study aimed to develop machine learning-based models for early post-treatment reassessment of post-thrombolysis outcomes in AIS patients, thereby providing a reliable tool for early prognostic reassessment after thrombolysis.

**Methods:**

A total of 383 AIS patients who received intravenous thrombolysis between November 2024 and November 2025 were retrospectively enrolled and randomly assigned to a training set (*n* = 268) and a validation set (*n* = 115) in a 7:3 ratio. Univariate analysis was initially conducted to identify indicators associated with thrombolysis outcomes (*p* < 0.05). Least Absolute Shrinkage and Selection Operator (LASSO) regression was subsequently applied for feature selection. Based on the selected variables, three machine learning models—Random Forest (RF), Gradient Boosting Machine (GBM), and Support Vector Machine (SVM)—were developed, with logistic regression established as a benchmark. Model performance was assessed using the area under the receiver operating characteristic curve (AUC), calibration curves, and decision curve analysis. Model interpretability was further evaluated using SHapley Additive exPlanations (SHAP) values.

**Results:**

Baseline characteristics were well-balanced between groups (all *p* > 0.05). Multivariate logistic regression identified a higher admission National Institutes of Health Stroke Scale (NIHSS) score, elevated admission blood glucose, a higher 24-h post-thrombolysis NIHSS score, increased infarct core volume, higher glycated hemoglobin and an elevated Neutrophil-to-Lymphocyte Ratio as significant risk factors for poor outcomes, whereas a larger ischemic penumbra volume emerged as a protective factor (*p* < 0.05). The RF model demonstrated superior predictive performance, achieving an AUC of 0.784 in the training set, along with improved calibration and greater clinical net benefit within the 0.0–0.60 risk threshold range compared to the GBM and SVM. SHAP analysis showed the importance ranking of core predictors.

**Conclusion:**

The proposed prediction model demonstrates satisfactory performance in estimating functional outcomes following thrombolysis in AIS patients. The identified core variables may offer valuable insights for clinical prognosis and the design of individualized thrombolytic strategies.

## Introduction

Acute ischemic stroke (AIS) is a leading cerebrovascular disease globally, associated with high mortality and long-term disability. Characterized by acute onset, rapid progression, and highly variable prognoses, AIS imposes a substantial burden on families and society (1–3). Intravenous thrombolysis, a first-line core treatment for AIS, effectively dissolves thrombi, restores cerebral perfusion, and significantly improves functional outcomes (1, 4, 5). However, due to individual differences in patient age, stroke severity, comorbidities, and pathophysiological status, some patients may still experience poor outcomes such as functional dependence or symptomatic intracranial hemorrhage after thrombolysis. Consequently, accurate prediction of prognosis following thrombolysis is crucial for optimizing individualized treatment strategies and balancing therapeutic benefits against potential risks (6, 7).

Traditional prognostic prediction often relies on univariate analysis or conventional statistical methods like logistic regression. These approaches struggle to capture the complex nonlinear relationships among clinical variables, leading to limited predictive performance. In recent years, machine learning algorithms, with their robust feature extraction and pattern recognition capabilities, have been widely applied in predicting outcomes after AIS thrombolysis. By integrating multidimensional data, including clinical, imaging, and laboratory parameters, these methods have significantly enhanced the discrimination and reliability of predictive models. Numerous studies have explored the value of machine learning models in this domain, providing important references for clinical prognostic assessment. For instance, Luo et al. conducted a multicenter prospective cohort study, integrating clinical data with biomarkers such as neuron-specific enolase and S100 calcium-binding protein *β*, and employed a LightGBM model to predict 3-month outcomes, achieving good predictive performance (8). Yu et al. focused on imaging innovation by constructing a predictive nomogram based on ischemic core growth rate, offering a unique imaging perspective for outcome prediction (9). Wang et al. targeted a specific population of patients aged ≥80 years, developing a predictive model centered on the National Institutes of Health Stroke Scale (NIHSS) score and admission blood glucose, thereby addressing prognostic assessment needs in this special group (10). Bu et al. concentrated on the key outcome of hemorrhagic transformation after thrombolysis, incorporating inflammatory markers like the Neutrophil-to-Lymphocyte Ratio (NLR) and comparing the performance of various machine learning models (11). Liu et al. constructed a model using data from two centers, selecting relevant indicators such as age and clinical examinations, providing a foundation for multicenter validation (12). Chen et al. investigated the associations between admission random blood glucose, fasting blood glucose, stress hyperglycemia ratio and functional outcomes in acute ischemic stroke patients treated with intravenous thrombolysis, and confirmed that the stress hyperglycemia ratio calculated by fasting blood glucose could independently predict poor 3-month functional outcomes, offering a more precise indicator for prognostic evaluation of glucose-related markers (13). Sarraj et al. clarified in a study of acute ischemic stroke patients with large ischemic core that ischemic core volume and ischemic penumbra status directly affect treatment benefits and clinical outcomes, verifying the key role of imaging indicators in stroke prognostic assessment (14). Sharma et al. summarized the immunopathological mechanism of neutrophil-lymphocyte ratio in acute ischemic stroke through a systematic review, confirming that this index can reflect the degree of post-stroke inflammatory response and is closely related to hemorrhagic transformation, neurological deterioration and long-term poor prognosis after intravenous thrombolysis (15). These studies have offered diverse perspectives on predicting outcomes following AIS intravenous thrombolysis. Building upon this research foundation, the present study aims to further explore predictive models for outcomes after AIS intravenous thrombolysis. By integrating routine clinical indicators, imaging features, and laboratory tests, this study will employ Random Forest (RF), Gradient Boosting Machine (GBM), and Support Vector Machine (SVM) algorithms. Through a process of feature selection and model training, we will evaluate the models’ discrimination, calibration, and clinical utility. The ultimate goal is to provide clinicians with a post-thrombolysis early reassessment tool to accurately evaluate patient prognosis during the early course and inform individualized treatment decisions.

## Materials and methods

### Sample size estimation

Based on previous similar studies, sample size was estimated using an incidence rate of 35% as a reference (8, 9, 12). This single-center retrospective study was designed with *α* = 0.05 (two-tailed) and *β* = 0.20 (power = 80%), planning to include 6–8 core predictive variables. According to the 10 events per variable principle for clinical prediction models, a maximum of 8 variables requires at least 80 positive events to ensure model stability. The minimum required sample size was calculated to be approximately 229 patients. After adjusting for a 5% loss to follow-up rate, the minimum sample size was revised to approximately 241 patients. A total of 427 patients were initially screened, and 44 patients were excluded due to hemorrhagic stroke (*n* = 8), missing key variables required for model development (*n* = 21), failure to complete 3-month follow-up (*n* = 12) and other exclusion criteria (*n* = 3). Missing data were handled by direct exclusion without imputation. This study ultimately enrolled 383 AIS patients who received intravenous thrombolysis between November 2024 and November 2025 were retrospectively enrolled and randomly assigned to a training set (*n* = 268) and a validation set (*n* = 115) in a 7:3 ratio. This split is a widely adopted convention in clinical machine learning studies for internal validation, providing sufficient data for stable model training while preserving an independent sample for preliminary performance evaluation.

To assess the pattern of missing data, we quantified missingness per variable among the 427 initially screened patients. The proportion of missing values was low across all core variables: admission NIHSS score (0.7%, *n* = 3), admission blood glucose (1.2%, *n* = 5), 24-h post-thrombolysis NIHSS score (1.4%, *n* = 6), infarct core volume (1.9%, *n* = 8), ischemic penumbra volume (2.1%, *n* = 9), HbA1c (1.6%, *n* = 7), and NLR (1.2%, *n* = 5). No variable exceeded 3% missingness. We further compared the baseline characteristics of the 21 patients excluded due to missing key variables with those of the 383 patients included in the final analysis. The excluded patients were similar to the included study patients in terms of age (61.9 ± 10.2 vs. 63.1 ± 9.1, *p* = 0.557), sex (male: 52.4% vs. 54.6%, *p* = 0.839), and admission NIHSS score (8.9 ± 3.8 vs. 9.1 ± 3.5, *p* = 0.778). No statistically significant differences were observed in other baseline variables (all *p* > 0.05), suggesting that missingness was unlikely to introduce substantial selection bias. Given the low missing proportion (<3% for all core variables) and balanced baseline characteristics between included and excluded patients, missing data were assumed to be missing at random (MAR), and complete case analysis was deemed appropriate for this study.

### Inclusion criteria

(1) Diagnosis of AIS confirmed by neuroimaging, consistent with the latest AIS management guidelines, and exclusion of intracranial hemorrhage (13). (2) Receipt of intravenous thrombolysis within 4.5 h of symptom onset, with the treatment regimen adhering to clinical practice guidelines, absence of absolute contraindications, and completion of the full thrombolytic therapy course. (3) Adult patients (≥18 years old) with AIS who underwent intravenous thrombolysis between November 2024 and November 2025. (4) Complete records of baseline clinical data, imaging characteristics, laboratory tests, and intravenous thrombolysis-related information, allowing for the extraction of all variables necessary for model development. (5) Availability of follow-up data on functional outcomes at 3 months (±7 days) post-thrombolysis.

### Exclusion criteria

(1) Confirmation of hemorrhagic stroke or transient ischemic attack on neuroimaging, without a definitive AIS diagnosis. (2) Patients with onset-to-treatment time >4.5 h, presence of absolute contraindications to intravenous thrombolysis, or failure to complete the full course of thrombolytic therapy. (3) Missing or incomplete records for key variables required for model development, including baseline clinical data, imaging features, or laboratory parameters. (4) Inability to complete the 3-month functional outcome follow-up for any reason. (5) Presence of major comorbidities that could confound the prognostic assessment of AIS thrombolysis, such as severe malignant tumors, end-stage organ failure, or other serious neurological diseases. (6) Patients who received endovascular thrombectomy, either as bridging therapy (intravenous thrombolysis followed by thrombectomy) or as rescue treatment, were excluded from this study.

### Data collection

Based on principles of clinical relevance, data standardization, and feasibility, this study systematically collected baseline clinical data, imaging parameters, and laboratory findings potentially influencing outcomes after AIS thrombolysis. All data were extracted from standardized sources within the hospital, including electronic medical records, the Picture Archiving and Communication System (PACS), and the laboratory information system, to ensure accuracy and completeness. Specific collected items and measurement methods are detailed below.

Baseline clinical data encompassed demographic characteristics, medical history, clinical signs at admission, and thrombolysis-related information. Age and sex were extracted directly from medical records. Body Mass Index (BMI) was calculated using the formula: weight/height^2^ (kg/m^2^). History of hypertension, diabetes mellitus, smoking, and alcohol consumption were confirmed based on medical records and patient self-report. Systolic and diastolic blood pressures at admission were the first measurements obtained using an electronic sphygmomanometer prior to thrombolysis. Admission NIHSS scores and 24-h post-thrombolysis NIHSS scores were assessed independently by trained neurologists according to the standardized NIHSS scale. Trial of Org 10,172 in Acute Stroke Treatment (TOAST) classification was determined by specialized physicians integrating clinical data and imaging findings. Onset-to-treatment time was precisely recorded as the duration from symptom onset to the initiation of thrombolytic agent infusion. The dose of the thrombolytic agent administered was recorded as per clinical practice.

Imaging parameters were obtained by specialized radiologists from the PACS based on non-contrast head CT, MRI, and angiography. Infarct core volume and ischemic penumbra volume were measured and calculated from post-processed CT/MRI images using dedicated imaging analysis software. The presence of large vessel occlusion was determined based on CT angiography or MR angiography findings, assessing for occlusion in major intracranial vessels such as the internal carotid artery and middle cerebral artery.

Laboratory parameters were measured from fasting venous blood samples collected prior to thrombolysis, following standard clinical laboratory protocols. Admission glucose, glycated hemoglobin (HbA1c), serum creatinine, and low-density lipoprotein cholesterol were measured using standardized automated biochemical analyzers. Platelet count was determined using an automated hematology analyzer. The NLR was calculated using the formula: neutrophil count / lymphocyte count, derived from the complete blood count.

### Outcome definition

The primary outcome of this study was the 3-month functional outcome after intravenous thrombolysis for AIS. According to the 2026 AIS management guidelines, functional outcome was uniformly assessed using the internationally recognized modified Rankin Scale (mRS) (16). This scale evaluates a patient’s level of disability and dependence in daily activities, grading outcomes into 7 levels from 0 to 6: 0, no symptoms; 1, symptomatic but no significant disability, able to perform all usual duties and activities; 2, slight disability, unable to perform all previous activities but able to look after own affairs without assistance; 3, moderate disability, requiring some help but able to walk without assistance; 4, moderately severe disability, unable to walk and attend to bodily needs without assistance; 5, severe disability, bedridden, incontinent, requiring constant nursing care; 6, dead.

For this study, a favorable functional outcome was defined as mRS score ≤2 (indicating patients who are independent or only slightly disabled, aligning with the primary goal of thrombolytic therapy). A poor functional outcome was defined as mRS score ≥3 (indicating moderate to severe disability, dependence, or death, representing patients requiring significant clinical attention).

Outcome assessment was performed at 3 months (±7 days) post-thrombolysis. Face-to-face evaluations were prioritized during outpatient follow-up visits. For patients unable to attend in person, a standardized telephone interview questionnaire was used to determine the mRS score. All assessors underwent uniform training and were blinded to the patients’ baseline clinical data to minimize assessment bias.

### Statistical analysis

Statistical analyses were performed using SPSS 26.0 and R 4.5.1. Graphs were generated using GraphPad Prism 9.0. The significance level was set at *α* = 0.05, with a two-tailed *p* < 0.05 considered statistically significant. Normality of continuous variables was tested. Normally distributed data were presented as mean ± standard deviation (X ± S), and comparisons between groups were made using the *t*-test. Non-normally distributed data were presented as median (interquartile range), and comparisons between groups were made using the Mann–Whitney U test. Categorical variables were presented as counts (percentages), and comparisons between groups were made using the Chi-square (*χ*^2^) test; Fisher’s exact test was applied when expected frequencies were <5.

Patients were randomly assigned to a training set and a validation set in a 7:3 ratio using a random number table. To ensure model stability and reproducibility, data preprocessing was performed first: continuous variables were standardized using Z-score normalization, and categorical variables were encoded with one-hot encoding. All feature selection procedures were implemented only in the training set to prevent data leakage. In the training set, univariate analysis was performed to identify potential predictive factors (*p* < 0.05). Pearson correlation analysis was performed to exclude highly redundant variables with correlation coefficient |*r*| > 0.7. Least Absolute Shrinkage and Selection Operator (LASSO) regression was then applied with 10-fold cross-validation to determine the optimal penalty parameter (lambda.1se) for core predictor selection. The selected variables were subsequently included in a multivariate logistic regression model using a stepwise backward elimination method. Based on the same core predictors, three machine learning models (RF, GBM, SVM) were trained solely on the training set, and the independent validation set was used only for final model evaluation without any involvement in feature selection or parameter tuning, completely avoiding data leakage. For model training, the RF model was set with 500 decision trees as a fixed parameter, while the number of features considered at each split was optimized via grid search over the range of 2 to 7. The GBM model adopted a fixed learning rate of 0.1 and 1,000 iterations, with the tree depth (interaction.depth) optimized over {1, 2, 3, 4} and the minimum number of observations per terminal node (n.minobsinnode) over {5, 10, 15}. The SVM model used a radial basis function kernel with a fixed gamma value of 1/n_features, and the cost parameter C was optimized over {0.1, 1, 10, 100, 1,000}. Hyperparameter optimization was performed via 10-fold cross-validation in the training set using the one-standard-error rule for final selection. To ensure robust model evaluation and mitigate the impact of random sampling bias, the entire model development and validation pipeline was repeated 5 independent times using different random seeds. In each run, patients were randomly assigned to a training set and a validation set in a 7:3 ratio using a random number table. All feature selection procedures and hyperparameter optimization were performed exclusively in the training set of each run to prevent data leakage. Final performance metrics reported in the Results section represent the mean values across the 5 independent runs, and standard deviations are provided for all validation set metrics to demonstrate the stability of model performance. For classification into poor vs. good outcome, the conventional default probability threshold of 0.5, which is widely used in binary classification machine learning studies, was used to derive sensitivity, specificity, positive predictive value, and negative predictive value. A nomogram model was constructed using the “rms” package in R, and variance inflation factor (VIF) was used to test the multicollinearity of variables in the nomogram.

Receiver operating characteristic (ROC) curves were plotted using GraphPad Prism 9.0, and the Area under the ROC curve (AUC) with its 95% CI was calculated to assess the discrimination ability of all models. Calibration curves were plotted, the Hosmer-Lemeshow goodness-of-fit test and mean absolute error were used to assess the agreement between predicted probabilities and actual outcomes as well as the calibration accuracy of the models. Decision curve analysis (DCA) was performed to evaluate the clinical net benefit of each model at different risk thresholds. SHapley Additive exPlanations (SHAP) analysis was applied to quantify the feature importance of core predictors and interpret the machine learning models. Internal validation was performed using 1,000 bootstrap resamples to correct for overfitting bias.

## Results

### Comparison of baseline characteristics between training and validation sets

Among the 268 patients in the training set, 88 (32.84%) experienced a poor outcome following intravenous thrombolysis. In the validation set (*n* = 115), 37 patients (32.17%) had a poor outcome. No statistically significant differences were observed in baseline characteristics between the training and validation sets (all *p* > 0.05) (Table 1).

**Table 1:** Comparison of baseline characteristics between training and validation sets.

Characteristic		Training set (*n* = 268)	Validation set (*n* = 115)	*t/χ^2^*	*p*
Age (years)		62.87 ± 9.30	63.58 ± 8.22	0.703	0.482
Sex	Male	144 (53.73)	65 (56.52)	0.253	0.615
	Female	124 (46.27)	50 (43.48)		
Admission SBP (mmHg)		142.28 ± 17.39	139.54 ± 18.84	1.378	0.169
Admission DBP (mmHg)		83.59 ± 10.63	84.54 ± 9.84	0.819	0.413
BMI (kg/m^2^)		24.75 ± 2.95	24.45 ± 2.82	0.924	0.356
Admission NIHSS score		9.11 ± 3.62	9.24 ± 3.15	0.335	0.738
Admission glucose (mmol/L)		6.85 ± 1.38	6.95 ± 1.46	0.639	0.523
24-h post-thrombolysis NIHSS score		4.44 ± 2.31	4.21 ± 2.26	0.899	0.369
History of hypertension	Yes	146 (54.48)	68 (59.13)	0.707	0.401
	No	122 (45.52)	47 (40.87)		
History of diabetes	Yes	100 (37.31)	53 (46.09)	2.582	0.108
	No	168 (62.69)	62 (53.91)		
History of smoking	Yes	128 (47.76)	50 (43.48)	0.593	0.441
	No	140 (52.24)	65 (56.52)		
History of alcohol intake	Yes	71 (26.49)	40 (34.78)	2.687	0.101
	No	197 (73.51)	75 (65.22)		
TOAST classification	LAA	173 (64.55)	70 (60.87)	1.982	0.576
	CE	47 (17.54)	25 (21.74)		
	SAA	41 (15.30)	15 (13.04)		
	Other	7 (2.61)	5 (4.35)		
Onset-to-treatment time (h)		2.70 ± 0.82	2.68 ± 0.75	0.224	0.823
Infarct core volume (cm^3^)		20.16 ± 9.20	19.85 ± 8.88	0.305	0.760
Large vessel occlusion	Yes	116 (43.28)	44 (38.26)	0.835	0.361
	No	152 (56.72)	71 (61.74)		
Ischemic penumbra volume (cm^3^)		17.84 ± 9.02	18.45 ± 8.74	0.612	0.541
HbA1c (%)		6.72 ± 1.37	6.88 ± 2.01	0.903	0.367
NLR		2.65 ± 1.34	2.71 ± 1.25	0.410	0.682
Platelet count (×10^9^/L)		219.62 ± 53.40	222.15 ± 41.65	0.452	0.651
Serum creatinine (μmol/L)		83.64 ± 20.83	80.54 ± 19.14	1.352	0.177
LDL-C (mmol/L)		2.98 ± 0.72	3.05 ± 0.57	0.925	0.355
Thrombolytic drug dose (mg)		6.21 ± 1.01	6.14 ± 1.04	0.616	0.538

### Univariate analysis of poor outcomes following intravenous thrombolysis in AIS patients

Univariate analysis of the training set showed significant differences between the poor outcome group and the good outcome group in admission NIHSS score, admission blood glucose, 24-h post-thrombolysis NIHSS score, infarct volume, ischemic penumbra volume, HbA1c, and NLR (all *p* < 0.05) (Supplementary Table 1).

### Multivariable logistic regression analysis of poor outcomes following intravenous thrombolysis in AIS patients

Pearson correlation analysis was preformed to evaluate the collinearity among variables with *p* < 0.05 in the univariate analysis for avoiding redundancy, which revealed a strong positive correlation between admission NIHSS score and 24-h post-thrombolysis NIHSS score (*r* = 0.68, *p* < 0.001), and a moderate positive correlation between admission blood glucose and HbA1c (*r* = 0.54, *p* < 0.001). With functional outcome as the dependent variable (poor outcome group = 1, good outcome group = 0), variables with *p* < 0.05 in the univariate analysis were included in a LASSO regression for variable selection, using the lambda.1se criterion (Supplementary Figure 1). The selected variables were then incorporated into a multivariable Logistic regression analysis. The results identified admission NIHSS score, admission blood glucose, 24-h post-thrombolysis NIHSS score, infarct volume, HbA1c, and NLR as independent risk factors for poor outcomes, while ischemic penumbra volume emerged as a protective factor (*p* < 0.05) (Table 2).

**Table 2:** Multivariable logistic regression analysis of poor outcomes following intravenous thrombolysis in AIS patients.

Factor	*β*	SE	Wald	*p*	OR	95%CI
Admission NIHSS score	0.105	0.043	5.913	0.015	1.11	1.021–1.208
Admission blood glucose	0.316	0.115	7.566	0.006	1.372	1.095–1.718
24-h post-thrombolysis NIHSS score	0.166	0.066	6.209	0.013	1.18	1.036–1.344
Infarct volume	0.042	0.017	6.358	0.012	1.043	1.009–1.078
Ischemic penumbra volume	−0.038	0.018	4.32	0.038	0.963	0.929–0.998
HbA1c	0.328	0.117	7.842	0.005	1.389	1.104–1.748
NLR	0.297	0.11	7.337	0.007	1.346	1.086–1.668

### Predictive performance of machine learning models in the training and validation sets

**Figure 1:**
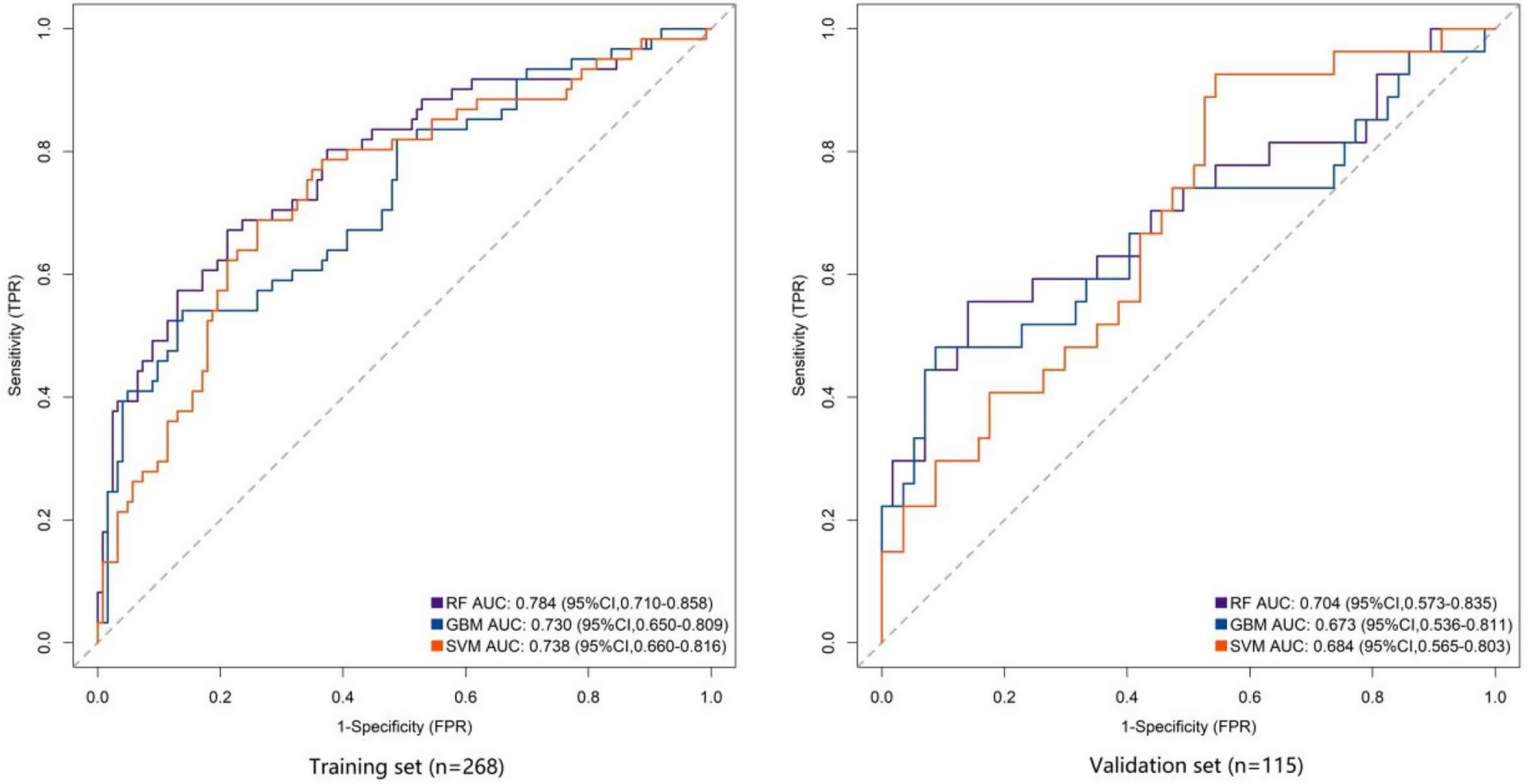
Receiver operating characteristic curve analysis of the machine learning models for predicting outcomes after intravenous thrombolysis.

**Figure 2:**
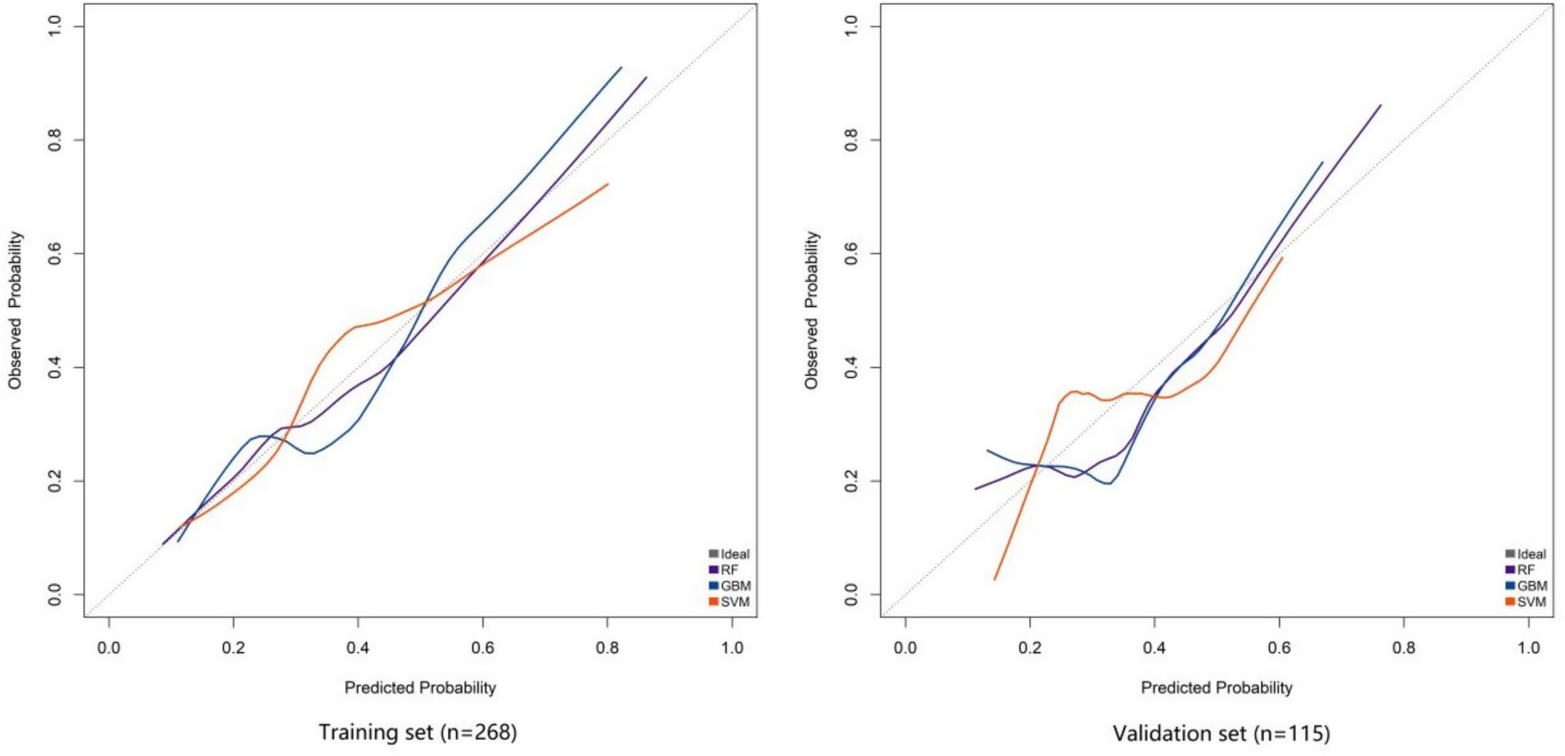
Calibration curve analysis of the machine learning models for predicting outcomes after intravenous thrombolysis.

ROC curves were plotted to evaluate the predictive performance of the three machine learning models (RF, GBM, and SVM) in the training and validation sets. In the training set (*n* = 268), the AUC values with 95% confidence intervals were 0.784 (95%CI: 0.710–0.858) for RF, 0.730 (95%CI: 0.660–0.809) for GBM, and 0.738 (95%CI: 0.660–0.816) for SVM. In the validation set (*n* = 115), the corresponding AUC values with 95%CI were 0.704 (95%CI: 0.573–0.835) for RF, 0.673 (95%CI: 0.536–0.811) for GBM, and 0.684 (95%CI: 0.565–0.803) for SVM (Figure 1). The standard deviations of the validation set AUC across runs were 0.021 for RF, 0.024 for GBM, and 0.019 for SVM, indicating stable and consistent performance across different data splits. Similar stability was observed for GBM (mean 0.670, SD 0.024) and SVM (mean 0.679, SD 0.019). Using a classification threshold of 0.5, the mean threshold-dependent performance metrics for all models in the validation set were calculated as follows. For the four models (RF, GBM, SVM, and logistic regression), the sensitivity was 0.703, 0.649, 0.676, and 0.622, respectively; the specificity was 0.685, 0.667, 0.659, and 0.689, respectively; the positive predictive value was 0.621, 0.585, 0.595, and 0.605, respectively; the negative predictive value was 0.754, 0.724, 0.731, and 0.705, respectively; and the Brier score was 0.182, 0.188, 0.197, and 0.195, respectively. Among these models, the RF model achieved the highest sensitivity and negative predictive value, which is critical for identifying high-risk patients who require intensified monitoring and intervention. It also had the lowest Brier score, indicating the best overall prediction accuracy. Calibration curves demonstrated that the RF model’s curve was closest to the diagonal line in the training set (Figure 2). While the Hosmer-Lemeshow test indicated adequate fit for all three models (*p* > 0.05), the RF model exhibited the highest calibration accuracy in both the training and validation sets. This suggests that the RF model’s predictions have smaller deviations from the actual outcomes and provide more stable calibration (Table 3). As the benchmark model, logistic regression achieved an AUC of 0.712 (95%CI: 0.638–0.786) in the training set and 0.665 (95%CI: 0.527–0.803) in the validation set.

**Table 3:** Calibration results for the three models in the training and validation sets.

Dataset	Model	Mean absolute error	Hosmer-Lemeshow Test	
			χ^2^	*P*
Training set	RF	0.166	3.709	0.882
	GBM	0.186	13.271	0.103
	SVM	0.186	6.522	0.589
Validation set	RF	0.183	12.270	0.140
	GBM	0.188	10.550	0.288
	SVM	0.197	8.759	0.363

### Decision curve analysis of machine learning models

**Figure 3:**
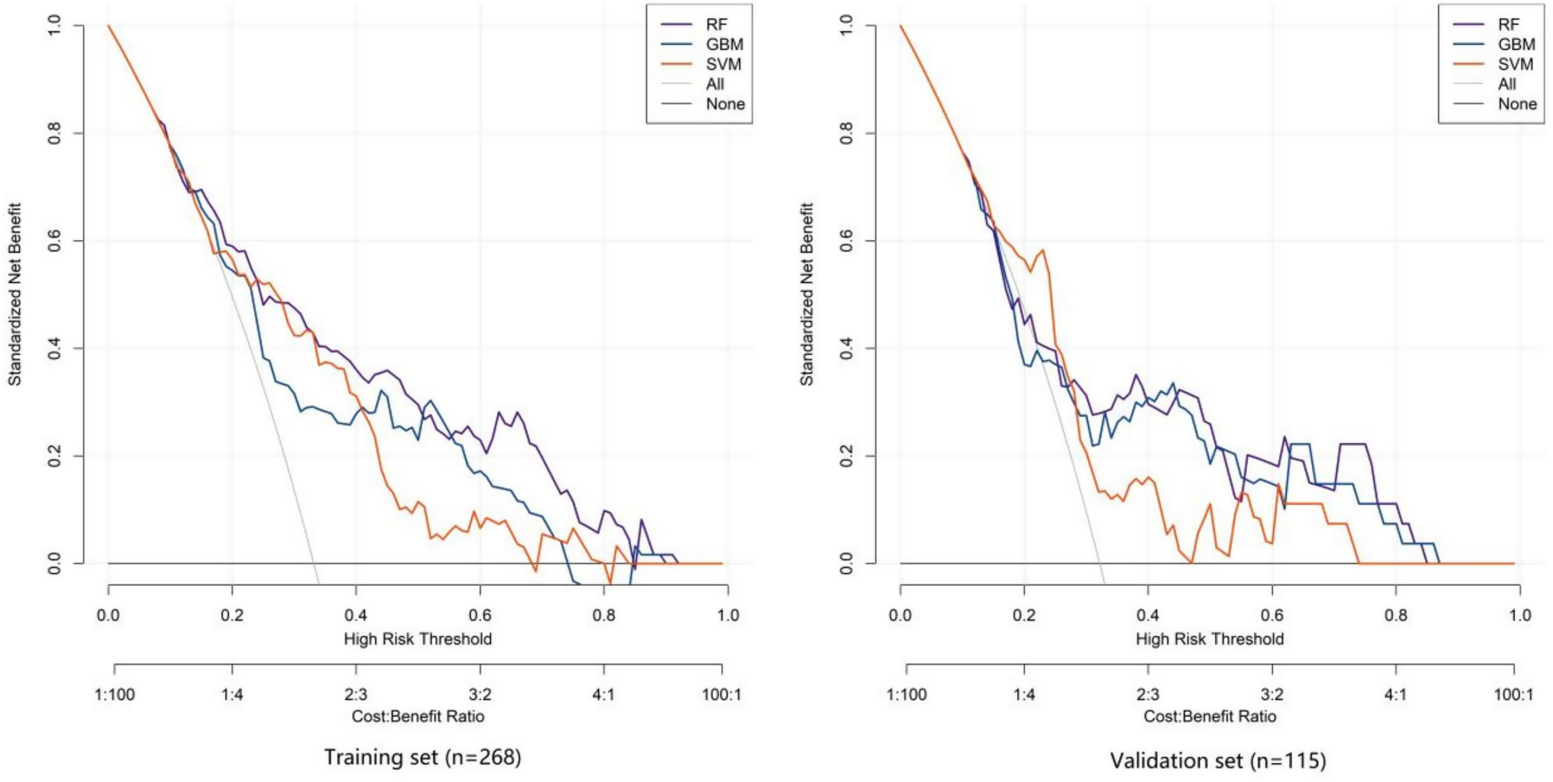
Decision curve analysis of the machine learning models for predicting outcomes after intravenous thrombolysis.

DCA showed that across both the training and validation sets, the RF model provided a higher net clinical benefit compared to the GBM and SVM models across most high-risk threshold probabilities, particularly within the clinically relevant range of 0 to 0.6. This benefit was also superior to the extreme strategies of treating all patients or treating none. In higher threshold regions, the net benefit of the RF model remained comparatively favorable, indicating its greater potential for guiding clinical decisions and delivering tangible patient benefit (Figure 3).

### Predictive model construction

**Figure 4:**
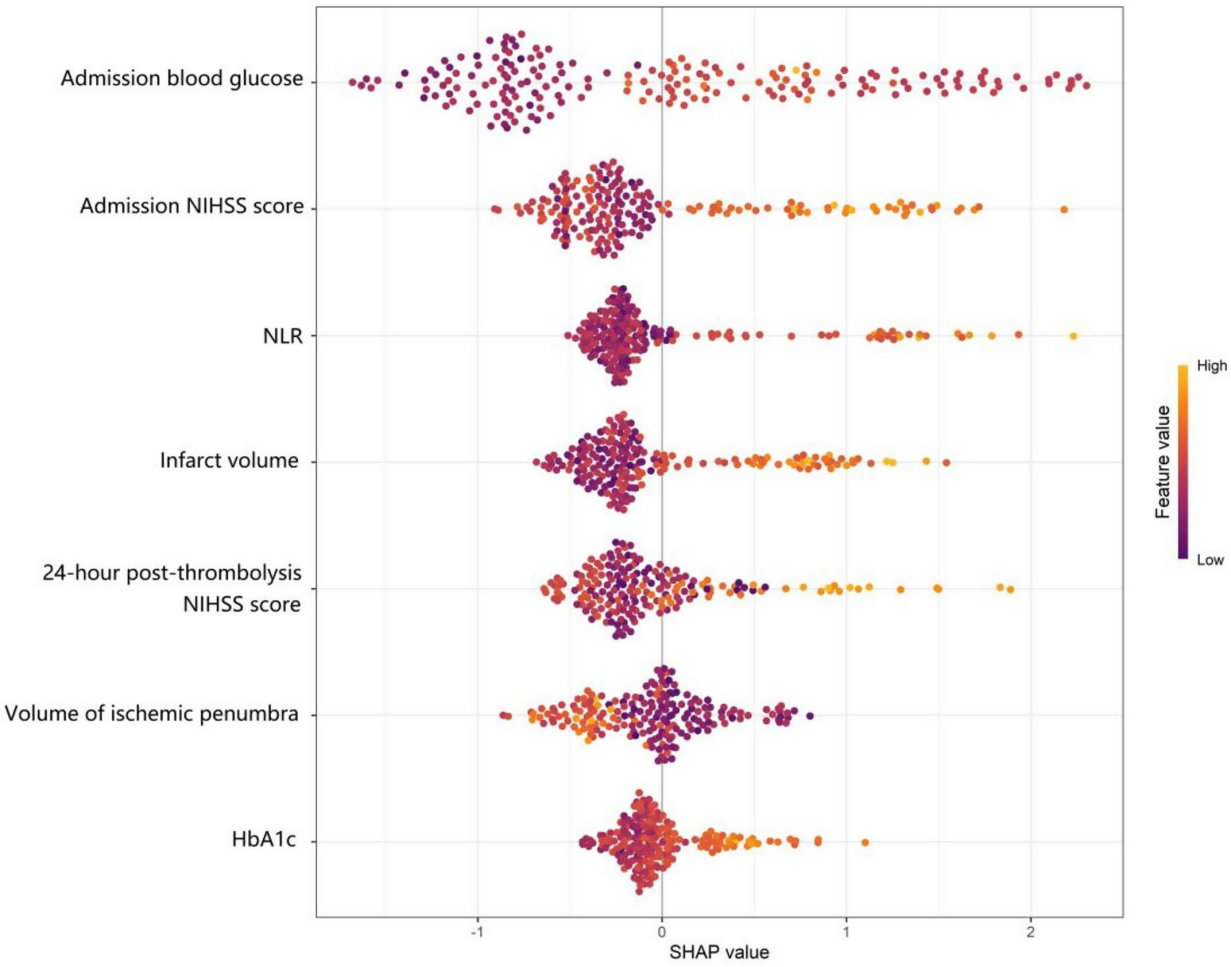
SHAP variable importance plot.

The error rate plot for the RF model showed a rapid decrease in the out-of-bag (OOB) error rate, which ultimately stabilized at a very low level. This reflects the strong generalization ability of the RF model, maintaining a low and stable error rate even when evaluated with OOB data (Supplementary Figure 2). SHAP variable importance plot indicated that the ranked importance of predictors was, in descending order: admission blood glucose, admission NIHSS score, NLR, infarct volume, 24-h post-thrombolysis NIHSS score, ischemic penumbra volume, and HbA1c (Figure 4).

### Nomogram model construction

**Figure 5:**
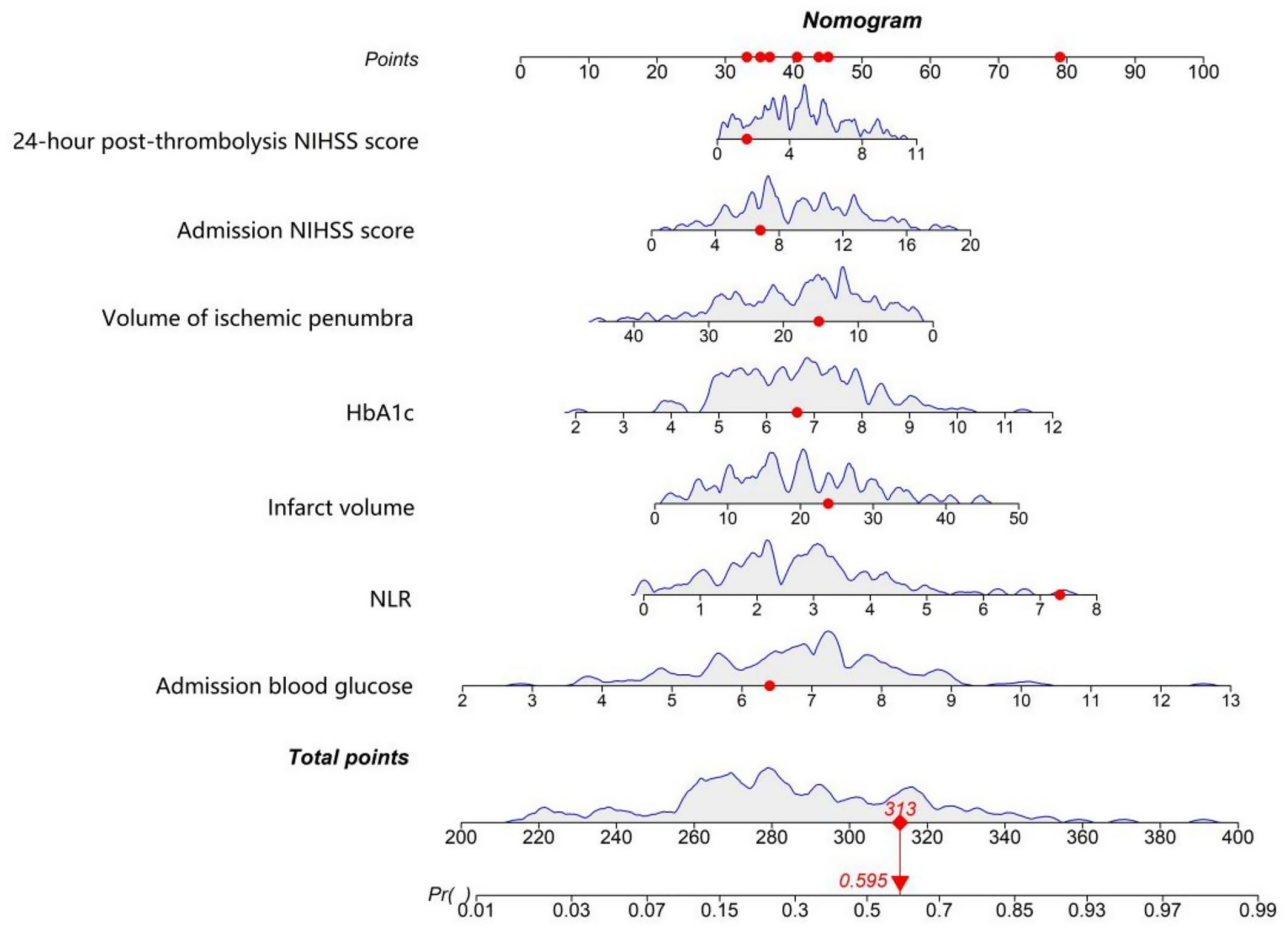
Nomogram for predicting poor outcome after intravenous thrombolysis in AIS patients.

A nomogram was developed based on the identified influencing factors to predict the outcome of intravenous thrombolysis in AIS. Each independent predictor was assigned a corresponding point value on the nomogram. The total score, calculated by summing the points for all predictors, allows for an estimation of the predicted outcome probability; a higher total score corresponds to a higher predicted probability of a poor outcome (Figure 5). Collinearity diagnostics showed VIFs of 1.069 for admission NIHSS score, 1.087 for admission blood glucose, 1.081 for 24-h post-thrombolysis NIHSS score, 1.022 for infarct volume, 1.060 for ischemic penumbra volume, 1.037 for HbA1c, and 1.027 for NLR. These low VIF values indicate negligible multicollinearity among the variables, suggesting that the linear relationships between the predictors are weak. Therefore, the adverse impact of multicollinearity on model estimation and inference is minimal, ensuring the stability and interpretability of the regression model.

The red dots on each variable axis of the nomogram represent the values for a sample patient who received intravenous thrombolysis for AIS (Figure 5). For instance, this patient had an admission NIHSS score of 7, a 24-h post-thrombolysis NIHSS score of 1, an ischemic penumbra volume of 16 cm^3^, an HbA1c of 6.55%, an infarct volume of 24 cm^3^, an NLR of 7.2, and an admission blood glucose of 6.3 mmol/L. To use the nomogram, a vertical line is drawn from the red dot on each variable axis to the top “Points” axis to obtain the corresponding points for each factor. These individual points are then summed to calculate a total score (313 points in this example, marked by the red dot on the “Total Points” axis). Finally, a vertical line is drawn from the total score on the “Total Points” axis down to the “Probability of Poor Outcome” axis to estimate the predicted probability. For this patient, the predicted probability of a poor 3-month functional outcome was 0.595, suggesting a high risk of an unfavorable prognosis post-thrombolysis and indicating a need for focused clinical attention and individualized intervention strategies.

## Discussion

This study identified the direction of association between various factors and poor outcomes following intravenous thrombolysis in AIS patients. Admission NIHSS score, admission glucose level, 24-h post-thrombolysis NIHSS score, infarct core volume, HbA1c and NLR were identified as independent risk factors, whereas the volume of the ischemic penumbra was a protective factor, which deeply reveals the pathophysiological mechanisms of neurological deficit, glycometabolism disorder, inflammatory response and ischemic penumbra salvage affecting thrombolysis prognosis. Compared with previous studies that only focused on single clinical, imaging or laboratory indicators, this study integrated multi-dimensional conventional indicators to improve the comprehensiveness of prediction. Among the three machine learning models (RF, GBM, SVM) developed using core variables, the RF model demonstrated superior predictive performance, with better calibration and higher clinical net benefit than the other two models, which was consistent with the advantages of RF model in handling non-linear relationships and overfitting prevention in stroke prognosis prediction. Notably, the RF model achieved the highest sensitivity (0.703) and negative predictive value (0.754) among all models, indicating its strong ability to correctly identify patients at risk of poor outcomes and to reliably rule out those who are likely to have favorable recovery. This high sensitivity and NPV make the RF model particularly valuable for clinical screening, as it can effectively flag high-risk patients who require intensified neurological monitoring and early individualized interventions after thrombolysis. SHAP analysis further solved the “black box” problem of machine learning and clarified the importance ranking of these core variables. Furthermore, a nomogram constructed from the core variables provided a visualized and quantitative tool for clinical rapid prognosis assessment, which is more applicable in clinical practice than pure model algorithms.

The seven core variables identified in this study are all closely associated with functional outcomes in AIS patients after intravenous thrombolysis. These variables influence thrombolytic prognosis through distinct physiological and pathological mechanisms, serving as key indicators reflecting disease severity, metabolic status, and the extent of brain tissue injury. Admission NIHSS score directly reflects the degree of neurological deficit upon admission; a higher score indicates more extensive and severe ischemic brain injury, where damaged neurons are less likely to recover rapidly after thrombolysis, thereby increasing the probability of poor outcomes (17, 18). Admission glucose and hemoglobin A1c reflect immediate and long-term glycemic status, respectively. Hyperglycemia can exacerbate cerebral ischemia–reperfusion injury by activating oxidative stress and disrupting blood–brain barrier integrity, leading to aggravated cerebral edema and neuronal apoptosis. Chronic hyperglycemia can also impair cerebrovascular endothelial function, reducing the efficacy of recanalization after thrombolysis and hindering neurological recovery (13, 19). The 24-h post-thrombolysis NIHSS score directly reflects early neurological improvement following thrombolysis. A higher score suggests inadequate thrombus dissolution or secondary brain injury, indicating a lack of effective early neurological recovery and predisposing patients to long-term poor outcomes (20).

Infarct volume is directly correlated with the extent of irreversibly damaged brain tissue. A larger infarct volume signifies more extensive neuronal loss, making functional recovery difficult even if successful recanalization is achieved. Moreover, a large infarct can lead to complications such as elevated intracranial pressure and cerebral edema, further worsening the prognosis (21). The NLR, a quantitative marker of inflammation, reflects the degree of inflammatory activation following cerebral ischemia. An elevated NLR indicates excessive post-ischemic inflammation; extensive neutrophil infiltration releases various inflammatory cytokines that exacerbate reperfusion injury, while concurrent lymphopenia suggests an immunosuppressed state. These factors collectively disrupt the cerebral microenvironment and impede neural repair processes (15). The ischemic penumbra represents potentially salvageable brain tissue. A larger penumbral volume indicates a greater extent of tissue that could be rescued by timely reperfusion, potentially preventing its progression to irreversible infarction, thereby reducing neurological deficits and acting as a protective factor for favorable thrombolytic outcomes (14, 22).

This study integrated routinely available clinical, imaging, and laboratory parameters, overcoming the limitations associated with using single predictors. By developing and comparing multiple machine learning models, a well-suited predictive model was selected. The integration of SHAP analysis enhanced model interpretability, addressing the “black box” nature of some machine learning algorithms. Additionally, the core variables were translated into a practical nomogram, offering ease of use and applicability in clinical settings.

This study has several limitations. The evaluation framework does not support strong conclusions on model robustness due to the single-center retrospective design, the internal validation based on repeated random splits (though performed) still requires external multi-center confirmation, lack of external multi-center validation, and wide 95% CI of validation AUC (0.573–0.835), indicating imprecision and potential instability of performance estimates. As a single-center retrospective study without external multi-center validation, the generalizability of the models requires further verification. The evaluation of model performance relies primarily on core discrimination and clinical utility metrics such as AUC, calibration curves, and DCA, without supplementing the two classification metrics of accuracy and F1-score (precision and recall have been covered via PPV and sensitivity, respectively, as reported in the Results section). Accuracy and F1-score are not reported because they are highly threshold-dependent and can be misleading under class imbalance; we therefore prioritized threshold-independent metrics (AUC and calibration) alongside clinically intuitive threshold-based metrics (sensitivity and PPV) for primary interpretation. The included variables were limited to routine clinical parameters; novel indicators such as genetic markers or specific serum biomarkers were not incorporated, potentially omitting relevant predictive factors. The follow-up duration was restricted to 3 months post-thrombolysis, lacking data on long-term functional outcomes and thus limiting assessment of the model’s predictive value for long-term prognosis. Furthermore, the potential impact of post-thrombolysis interventions, such as antiplatelet therapy or circulation-enhancing treatments, on patient outcomes was not considered, which might introduce bias. Lastly, subgroup analyses based on age or baseline disease severity were not performed, and the predictive value of the models for specific populations warrants further investigation. Furthermore, because patients who received endovascular thrombectomy were excluded, the findings of this study are most directly applicable to clinical settings where intravenous thrombolysis is administered without routine bridging thrombectomy. Generalizability to centers with high rates of thrombectomy or to patient populations routinely treated with endovascular therapy requires further validation. Future studies should specifically evaluate the performance of similar models in study that include thrombectomy-treated patients. Additionally, although admission SBP and DBP were collected, they did not show significant differences between outcome groups in univariate analysis and were therefore excluded from the final predictive models. Blood pressure is a well-recognized prognostic factor in acute ischemic stroke, and its lack of association in this study may be due to several reasons: the relatively narrow range of blood pressure values in our study, the exclusion of patients undergoing thrombectomy (who may have more pronounced blood pressure fluctuations), or insufficient statistical power to detect a modest effect. Future studies with larger sample sizes and broader blood pressure ranges should re-evaluate the predictive role of blood pressure in thrombolysis outcomes.

At the clinical level, the core variables identified in this study could serve as reference targets for early prognostic assessment in AIS patients receiving thrombolysis. Clinicians could utilize these parameters to rapidly identify patients at high risk for poor outcomes, enabling the development of tailored intervention strategies and follow-up plans. The constructed nomogram could also serve as a practical tool to aid clinical decision-making. From a policy perspective, promoting the establishment and sharing of multi-center stroke databases is recommended. Standardizing data collection and management would provide a foundation for validating and refining prognostic models. Regarding future research, multi-center prospective studies are needed for external validation of the models constructed here. Incorporating additional dimensions, such as radiomics, genetic markers, and novel inflammatory factors, could enrich model features and enhance predictive accuracy. Extending the follow-up duration would allow assessment of the models’ value in predicting long-term functional outcomes. Subgroup analyses focusing on specific populations, such as elderly patients or those with multiple comorbidities, could lead to tailored prognostic models. Finally, further optimization of machine learning algorithms could improve model interpretability and ease of use, facilitating their implementation in primary healthcare settings.

## Conclusion

This retrospective analysis of clinical data from AIS patients treated with intravenous thrombolysis identified seven core variables associated with functional outcomes at 3 months post-treatment. Admission NIHSS score, admission glucose, 24-h post-thrombolysis NIHSS score, infarct volume, hemoglobin A1c, and NLR were identified as risk factors for poor thrombolytic outcomes, while ischemic penumbra volume served as a protective factor. Machine learning models based on these core variables demonstrated satisfactory discrimination, reliability, and clinical utility in predicting thrombolytic outcomes. SHAP analysis clarified the importance ranking of the core variables, and the constructed nomogram, free of significant multicollinearity and exhibiting good stability, enabled quantitative risk assessment. The identified core variables provide reference points for the early clinical identification of patients at high risk for poor outcomes and offer data to support individualized treatment strategies. Constrained by its single-center, retrospective design and lack of external multi-center validation, the generalizability of these models requires further confirmation. Subsequent multi-center prospective studies are necessary to validate and refine the models, incorporate additional variable dimensions, and ultimately enhance their predictive value, providing a more comprehensive reference for the clinical management of AIS patients undergoing intravenous thrombolysis outcomes.

## Data Availability

The original contributions presented in the study are included in the article/Supplementary material, further inquiries can be directed to the corresponding author.
