# Supplementary material for: Predicting intravenous thrombolysis outcomes in acute ischemic stroke using machine learning
Source: Front Neurol. 2026 Jul 23;17:1831043. doi: 10.3389/fneur.2026.1831043 (PMC13441995; doi:10.3389/fneur.2026.1831043)
Supplement: Supplementary file 1 [file Table_1.docx]

**Supplementary Figure 1.** LASSO coefficient profile plot (a) and cross-validation plot for tuning parameter selection (b)

**Supplementary Figure 2.** Error rate plot for the random forest model
