# Supplementary material for: Predicting intravenous thrombolysis outcomes in acute ischemic stroke using machine learning
Source: Front Neurol. 2026 Jul 23;17:1831043. doi: 10.3389/fneur.2026.1831043 (PMC13441995; doi:10.3389/fneur.2026.1831043)
Supplement: Supplementary file 2 [file Table_2.docx]

**Supplemental Table 1.** Univariate analysis of poor outcomes following intravenous thrombolysis in ais patients.

| Characteristic | | Poor Outcome Group (n=88) | Good Outcome Group (n=180) | *t/χ²* | *P* |
| --- | --- | --- | --- | --- | --- |
| Age (years) | | 63.45±9.62 | 62.58±9.15 | 0.719 | 0.473 |
| Sex | Male | 49(55.68) | 95(52.78) | 0.201 | 0.654 |
|  | Female | 39(44.32) | 85(47.22) |  |  |
| Admission SBP (mmHg) | | 143.25±17.85 | 141.81±17.20 | 0.640 | 0.523 |
| Admission DBP (mmHg) | | 84.10±10.95 | 83.35±10.50 | 0.541 | 0.589 |
| BMI (kg/m²) | | 24.65±3.05 | 24.80±2.90 | 0.391 | 0.696 |
| Admission NIHSS score | | 10.15±4.35 | 8.60±3.10 | 3.350 | 0.001 |
| Admission glucose (mmol/L) | | 7.25±1.45 | 6.65±1.30 | 3.415 | 0.001 |
| 24-hour post-thrombolysis NIHSS score | | 5.15±2.65 | 4.10±2.05 | 3.566 | 0.001 |
| History of hypertension | Yes | 46(52.27) | 100(55.56) | 0.257 | 0.612 |
|  | No | 42(47.73) | 80(44.44) |  |  |
| History of diabetes | Yes | 30(34.09) | 70(38.89) | 0.582 | 0.446 |
|  | No | 58(65.91) | 110(61.11) |  |  |
| History of smoking | Yes | 40(45.45) | 88(48.89) | 0.279 | 0.597 |
|  | No | 48(54.55) | 92(51.11) |  |  |
| History of alcohol intake | Yes | 26(29.55) | 45(25.00) | 0.627 | 0.428 |
|  | No | 62(70.45) | 135(75.00) |  |  |
| TOAST classification | LAA | 57(64.77) | 116(64.44) | 0.105 | 0.991 |
|  | CE | 15(17.05) | 31(17.22) |  |  |
|  | SAA | 13(14.77) | 28(15.56) |  |  |
|  | Other | 3(3.41) | 5(2.78) |  |  |
| Onset-to-treatment time (h) | | 2.75±0.85 | 2.68±0.80 | 0.659 | 0.511 |
| Infarct core volume (cm³) | | 23.25±10.10 | 18.65±8.35 | 3.947 | 0.001 |
| Large vessel occlusion | Yes | 36(40.91) | 80(44.44) | 0.301 | 0.583 |
|  | No | 52(59.09) | 100(55.56) |  |  |
| Ischemic penumbra volume (cm³) | | 15.55±7.10 | 18.95±9.65 | 2.938 | 0.004 |
| HbA1c (%) | | 7.05±1.45 | 6.55±1.30 | 2.846 | 0.005 |
| NLR | | 3.05±1.75 | 2.45±1.05 | 3.493 | 0.001 |
| Platelet count (×10⁹/L) | | 214.85±55.20 | 221.95±52.50 | 1.022 | 0.308 |
| Serum creatinine (μmol/L) | | 85.25±22.00 | 82.85±20.25 | 0.885 | 0.377 |
| LDL-C (mmol/L) | | 3.05±0.75 | 2.95±0.70 | 1.073 | 0.284 |
| Thrombolytic drug dose (mg) | | 6.15±1.05 | 6.25±1.00 | 0.756 | 0.450 |
